# Development of a multivariate prediction model for antidepressant resistant depression using reward-related predictors

**DOI:** 10.3389/fpsyt.2024.1349576

**Published:** 2024-03-25

**Authors:** Xiao Liu, Stephen J. Read

**Affiliations:** Department of Psychology, University of Southern California, Los Angeles, CA, United States

**Keywords:** depression, treatment-resistant, antidepressants, SSRI, anhedonia, internalizing, reward, machine learning

## Abstract

**Introduction:**

Individuals with depression who do not respond to two or more courses of serotonergic antidepressants tend to have greater deficits in reward processing and greater internalizing symptoms, yet there is no validated self-report method to determine the likelihood of treatment resistance based on these symptom dimensions.

**Methods:**

This online case-control study leverages machine learning techniques to identify differences in self-reported anhedonia and internalizing symptom profiles of antidepressant non-responders compared to responders and healthy controls, as an initial proof-of-concept for relating these indicators to medication responsiveness. Random forest classifiers were used to identify a subset from a set of 24 reward predictors that distinguished among serotonergic medication resistant, non-resistant, and non-depressed individuals recruited online (*N* = 393). Feature selection was implemented to refine model prediction and improve interpretability.

**Results:**

Accuracies for full predictor models ranged from .54 to .71, while feature selected models retained 3-5 predictors and generated accuracies of .42 to .70. Several models performed significantly above chance. Sensitivity for non-responders was greatest after feature selection when compared to only responders, reaching .82 with 3 predictors. The predictors retained from feature selection were then explored using factor analysis at the item level and cluster analysis of the full data to determine empirically driven data structures.

**Discussion:**

Non-responders displayed 3 distinct symptom profiles along internalizing dimensions of anxiety, anhedonia, motivation, and cognitive function. Results should be replicated in a prospective cohort sample for predictive validity; however, this study demonstrates validity for using a limited anhedonia and internalizing self-report instrument for distinguishing between antidepressant resistant and responsive depression profiles.

## Introduction

Major Depressive Disorder (MDD) is a heterogeneous disorder with widespread effects (1). Serotonergic antidepressants (e.g. selective serotonin reuptake inhibitors and serotonin and norepinephrine reuptake inhibitors; SSRIs and SNRIs) are standard first-line treatment for MDD, but have high non-response rates and a 6-8 week latency for symptom reduction (2, 3). There is currently no standard set of self-report items for prediction of response likelihood to SSRI/SNRIs in a clinical setting. Patients are often asked to complete extensive questionnaires and multiple self-report scales upon intake, which increases treatment and diagnostic burden. Therefore, we aim to identify a limited set of self-report items that can be administered with minimal burden to clinicians and patients for identifying pre-morbid treatment resistance to serotonergic antidepressants. In this study, we provide a proof-of-concept by first identifying a set of scales related to reward processing that differentiate between individuals with depression (MDD), antidepressant-resistant depression (ARD), and non-depressed adults. We intend to use this set of items in future research to determine their predictive validity for ARD.

Anhedonia is a symptom frequently present in individuals with depression following the administration of serotonergic antidepressants, and presence of anhedonia at pre-treatment predicts poorer response to these medications (4–11). Anhedonia arises from impairments in reward processing (12–14). It is defined in the *Diagnostic and Statistical Manual of Mental Disorders, Fifth Edition* (15) as low interest in and hedonic pleasure for reward. Alternative depression treatments such as esketamine and neuromodulatory therapies are used after non-response to multiple rounds of serotonergic medication has been established, and these treatments often specifically target anhedonia via the dopaminergic reward system (16–19). There is strong empirical evidence that traditional antidepressants such as SSRI/SNRIs can induce emotional blunting and apathy in individuals with depression (20–23). Fatigue and lack of concentration have also been reported as persistent residual symptoms post-treatment (24, 25), which can both be mechanistically linked to reward processing as they arise due to dopamine and norepinephrine deficiencies (26–28). Anhedonia in MDD is multifaceted (12), leading to a need for identifying the combination of anhedonia subcomponents with the greatest validity for discriminating between non-resistant MDD and ARD. We aim to balance discriminant validity with clinical utility by identifying a set of items that are practical to administer.

The National Institute of Mental Health has incorporated research delineating the function of reward processing into a framework of transdiagnostic neurobiological and behavioral mechanisms. This Research Domain Criteria framework posits that the domain of Positive Valence Systems is composed of reward responsiveness, reward valuation, and reward learning. These also map onto neural models of anticipatory vs. consummatory anhedonia proposed and validated by Berridge (29) such that anticipation maps onto “wanting” and consummation maps onto “liking”. Berridge found these processes to be governed by disparate brain networks and to operate somewhat independently of each other (29–31). Recent studies have presented a more detailed chain of neural signaling in reward processing: (1) incentive salience (internally cued desire; wanting), (2) anticipation (readiness for reward), (3) motivation (effort to obtain the reward), (4) hedonic response (consummation of reward, or liking), and (5) feedback integration (learning) (32–34). Additionally, personality traits such as extraversion have been shown to modulate sensitivity to reward (35).

In line with recent efforts to define the dimensional structure underlying psychopathology (36–38), we recognize anhedonia as part of a broader transdiagnostic endophenotype of internalizing symptomatology (39, 40). An internalizing spectrum of psychopathology has been well established and includes depressive disorders, general anxiety disorder, social anxiety disorder, and panic disorder, all of which are characterized by high levels of mood and cognitive disturbances (41–43). A common internalizing mechanism may help explain high rates of comorbidity between these disorders. Empirical research converges with a model of internalizing factors consisting of low positive affect in the form of loss of motivation and interest (anhedonia) and high negative affect in the form of anxious arousal and apprehension (39, 40). Thus, comprehensive measurement is needed to gain information about the type(s) of anhedonia and related impairments present in ARD.

To advance research, it is necessary to first identify where the greatest differences exist in individuals with ARD versus antidepressant responsive MDD, and how these vary from more general differences between individuals with and without depression. Machine learning methods have been increasingly used for complex biological models with limited sample sizes and have demonstrated utility in finding patterns, especially within high dimensional data (44–48). For a detailed account of the advantages to using machine learning methods over traditional regression, please see **Supplementary Materials**.

In the current paper, we rely on Random Forests, a non-parametric statistical technique. Non-parametric statistical techniques make no assumptions about the underlying distribution of the data, and similarly, non-parametric machine learning models do not assume a pre-specified form. Non-parametric classification algorithms have been used in large naturalistic MDD studies such as the Sequenced Treatment Alternatives to Relieve Depression (STAR*D), Combining Medications to Enhance Depression Outcomes (CO-MED), Genome-based Therapeutic Drugs for Depression (GENDEP) and the German Research Network on Depression (GRND) databases to predict treatment outcomes using sets of clinical and sociodemographic predictors, with reported accuracy rates ranging between.5 to.8 (44, 49–53). However, criticisms of using such models for treatment prognosis include their complexity, requiring comprehensive symptom and treatment data on each patient. In addition, they have a “black box” methodology where the process of prediction is either hidden or uninterpretable. Thus, machine learning techniques have been leveraged at the basic and translational research phases but require more simplification and transparency to be useful in clinical application. To date there has been limited work on using findings generated from basic and translational research to develop a practical instrument for predicting antidepressant medication prognosis.

We will use supervised machine learning methods to differentiate individuals who are ARD and medication responsive using a limited but comprehensive set of phenomenological predictors related to reward. We aim to provide an initial proof-of-concept for a practical self-report instrument to identify individuals with ARD based on a limited set of anhedonia and related items, which can then be refined and validated longitudinally. Additionally, we will use unsupervised machine learning to explore empirical patterns in the subset of significant predictors. To be useful in clinical practice, this proto-instrument will need to distinguish individuals with ARD from a population of potential patients who either (1) have depression or (2) do not have depression. Therefore, unlike previous machine learning studies that draw only from a population of patients with depression, this study will assess 3 groups of individuals: ARD, non-resistant depression (MDD), and non-depressed healthy controls (HC). In line with recent work using a wider range of clinical and sociodemographic variables for predicting treatment-resistance (52), it is hypothesized that we will be able to identify a set of measures and items to discriminate between groups at clinically meaningful levels (44, 54).

## Materials and methods

### Participants

The methods for this study, including sample size and analyses, were registered prior to viewing any collected data (55). Participants (N = 399, female_prop_ = .49) aged 18 or older were recruited using Prolific and ResearchMatch from a population pool within the United States between the months of April-December 2022. The number of participants deviated from the preregistered sample size of N = 600, although are achieved sample size is adequate for Random Forests. Recent evidence suggests a rule of thumb of 5 - 10 events per predictor variable, with the upper end recommended for samples with 30 events or fewer (56, 57). In this study, the event of interest was presence of ARD (N = 164), and classification models used up to 24 predictors, thus falling within in the acceptable range.

ResearchMatch is a national health volunteer registry funded by the U.S. National Institutes of Health as part of the Clinical Translational Science Award (CTSA) program. ResearchMatch volunteers have consented to be contacted by researchers about health studies for which they may be eligible. Prolific is an online research platform with behavioral and diagnostic filtering capabilities that helps researchers post studies and recruit from a general population. Our sample consisted of a similar proportion of participants recruited from Prolific (51%) and ResearchMatch (49%). The proportion of individuals within each group by platform is provided in **Supplementary Materials** (S0).

*Inclusion criteria* were adults fluent in English, who have a self-identified diagnosis of unipolar depression with either symptom improvement from at least 1 full course (> 4 weeks) of SSRI/SNRI medication) or non-improvement with at least 2 full courses of SSRI/SNRIs. Clinicians frequently use subjective report when defining depression treatment response (58). This study’s use of self-classification aligns with previous research methodology for identifying treatment resistance and uses the definition of inadequate response published by the US Food and Drug Administration (59) and European Medicines Agency (60). Clinical records were not obtained as we tried to minimize risk to participants of identification by collecting anonymous data. We additionally recruited a non-depressed control sample who have never been diagnosed with depression and scored **≤** 3 on the Patient Health Questionnaire-2 (PHQ-2) (61). *Exclusion criteria* were individuals with bipolar depression, psychosis, ADHD, and any personality disorder, to minimize confounding variables due to different treatments for these disorders. We also excluded individuals regularly taking bupropion, stimulant medications, pramipexole, or L-dopa medication due to their direct effects on the dopamine reward system. However, we did not exclude individuals on the basis of substances of abuse.

We recruited participants who were not treatment naïve so they could identify whether antidepressants worked for them. This was a cross sectional study aimed at investigating the differences in reward processing for individuals with and without ARD, and determining the predictive validity of these reward measures was out of the scope of this study. To confirm that minimal or no effects of serotonergic medication on anhedonia existed in our sample, we conducted moderation analyses for reported presence of medication on the effect of group on each anhedonia metric with the intent to exclude measures moderated by presence of medication.

Screening for individuals recruited from ResearchMatch was implemented online via the REDCap (Research Electronic Data Capture; 62, 63) platform hosted at the University of Southern California. On Prolific, screening was implemented via a study where participants were compensated based on average time spent. Screening measures included an author-constructed questionnaire of depression diagnosis and treatment history for recruitment of the 2 depression groups and the PHQ-2 (61) for recruitment of the healthy control group. Screening was also used to balance ARD vs. MDD groups. Review and approval for this study and all procedures was obtained from the institutional review board at the University of Southern California.

### Procedure

Participants were administered a battery of validated scales measuring depression, reward anticipation and hedonic experience, motivation, and personality via the online survey platform “Psytoolkit” (64, 65). This platform does not allow surveys to be saved and returned to at a later time. Survey items were grouped and displayed across 3 pages, and participants were told that they must reach the end of the study to be compensated. However, survey items were not mandatory and participants were able to navigate back and forth across pages. Validated scales were chosen to represent all stages of reward processing and stable traits related to reward. Scales were selected for inclusion if they contained items measuring a distinct component of anhedonia. Inter-item reliabilities for the scales are reported in **Table 1** and ranged from acceptable to high. Summary statistics of item means and standard deviations (SD) for each of the 24 subscales are displayed in **Table 2**.

**Table 1:** Inter-item reliabilities for each scale.

Scale	Cronbach’s α
PHQ-9	.87
MEI	.91
TEPS	.82
MASQ	.90
BFAS	.74
DASS	.97
BIS	.74
BAS	.85
ACIPS	.92

**Table 2:** Summary statistics of predictor means by group. Pre-imputed statistics are calculated from the non-missing items for each subscale.

Raw Data Summary Statistics by Diagnostic Group					
Predictor	HC, N = 100*^1^*	MDD, N = 129*^1^*	ARD, N = 164*^1^*	p-value*^2^*	q-value*^3^*
MEIme	7.32 (1.93)	6.10 (2.25)	5.13 (2.01)	<.001	<.001
MEIpe	6.18 (1.77)	4.38 (2.33)	4.54 (2.14)	<.001	<.001
MEIsm	8.35 (2.85)	6.83 (3.01)	7.04 (3.10)	<.001	.001
TEPSc	4.09 (.84)	4.34 (.91)	4.08 (.83)	.014	.023
TEPSa	3.84 (.67)	3.81 (.75)	3.59 (.76)	.052	.063
MASQaa	2.04 (.91)	2.15 (.89)	2.57 (.90)	<.001	<.001
MASQad	3.37 (.69)	3.77 (.87)	3.72 (.98)	<.001	<.001
MASQgd	2.54 (.77)	2.77 (.96)	3.21 (.81)	<.001	<.001
BFASnw	3.01 (.69)	3.35 (.77)	3.47 (.66)	<.001	<.001
BFASnv	2.77 (.77)	3.03 (.85)	3.08 (.73)	.008	.013
BFASee	3.00 (.66)	3.02 (.83)	2.72 (.65)	.004	.007
BFASea	2.94 (.69)	2.84 (.84)	2.92 (.75)	.500	.500
IDASdy	2.42 (.73)	2.77 (.93)	3.21 (.78)	<.001	<.001
IDASla	2.48 (.79)	3.01 (.89)	3.23 (.87)	<.001	<.001
DASSd	.97 (.67)	1.19 (.76)	1.59 (.73)	<.001	<.001
DASSa	.84 (.76)	.85 (.72)	1.18 (.68)	<.001	<.001
DASSs	1.06 (.69)	1.13 (.68)	1.47 (.62)	<.001	<.001
BIS	2.84 (.46)	3.00 (.47)	2.94 (.47)	.021	.030
BASd	2.45 (.61)	2.34 (.70)	2.54 (.64)	.025	.033
BASr	2.96 (.58)	3.02 (.65)	2.86 (.60)	.051	.063
BASf	2.65 (.55)	2.49 (.66)	2.63 (.65)	.200	.200
ACIPSgs	4.00 (1.08)	4.12 (1.34)	3.74 (1.16)	.021	.030
ACIPSis	3.99 (.90)	4.13 (1.07)	3.86 (.93)	.063	.072
ACIPSsb	3.88 (.94)	4.04 (1.11)	3.90 (1.01)	.200	.200

^1^Mean (SD).

^2^Kruskal-Wallis rank sum test.

^3^False discovery rate correction for multiple testing.

### Instrumentation

#### Depression measure

1. Patient health questionnaire-9 (PHQ-9) (66): 9 item 4-point Likert-based scale on frequency of depression symptomatology over the past 2 weeks. The PHQ-9 was validated against the mental health professional interview in a sample of 3000 patients, with a reported sensitivity of 75% and a specificity of 90% for major depression.

#### Reward processing measures

1. Inventory of depression and anxiety symptoms expanded version (IDAS-II) (67) The dysphoria subscale (IDASdy) contains items related to depressed mood, worthlessness, and guilt. The lassitude subscale (IDASla) contains items reflecting low energy. This study only administered the subset of items contained in these 2 subscales.
2. Temporal experience of pleasure scale (TEPS) (68): 18 item 6-point Likert-based scale consisting of two subscales measuring consummatory (TEPSc) and anticipatory (TEPSa) experience of pleasure.
3. Motivation and energy inventory (MEI) (69): 30-item Likert-based questionnaire with subscales for mental energy (MEIme), physical energy (MEIpe), and social motivation (MEIsm). The MEIme subscale is composed of cognitive functioning items, such as memory, concentration, and decision-making. The MEIpe subscale is composed of physical energy items. The MEIsm subscale is composed of items related to both interest and frequency of social activity and motivation for recreational activities. Each MEI subscale significantly distinguished between responders and non-responders in an 8-week antidepressant vs placebo trial (p <.001 for all pairwise t-tests).
4. Mood and anxiety symptoms questionnaire short adaptation (MASQ-D30) (70): 30-item 5-point Likert-based short form of the MASQ (71) used to assess trait symptomatology based on Clark and Watson’s Tripartite Model (41) of psychopathology, with 10 items each loading onto general distress (MASQgd), anxious arousal (MASQaa), and anhedonic depression (MASQad) factors.
5. Depression anxiety stress scale (DASS) (72): 42-item 4-point Likert-based scale designed to assess functioning using 3 subscales: depression (low positive affect and hopelessness; DASSd), anxiety (arousal and hyperarousal; DASSa), and stress (agitation or negative affect; DASSs).
6. Anticipatory and consummatory interpersonal pleasure scale (ACIPS) (73): 17 item 6-point Likert-based scale consisting of 7 anticipatory and 10 consummatory social pleasure items. Three subscales indicating anhedonia toward intimate social interactions (ACIPSis), group social interactions (ACIPSgs), and social bonding and making connections (ACIPSsb).

#### Trait measures

1. Behavioral inhibition activation scale (BIS/BAS) (74): 24 item 4-point Likert-based scale consisting of 4 subscales mapping onto behavioral inhibition (BIS), drive (BASd), reward responsiveness (BASr), and fun-seeking (BASf). The latter 3 were found to strongly load onto a second order factor of behavioral activation.
2. Big five aspect scale (BFAS) (75): 100-item 5-point Likert scale assessing two factor components of each of the big five personality constructs. In this study only items related to extraversion and neuroticism were used, as these traits are most related to a diathesis for depression. Extraversion is composed of the enthusiasm (BFASee) and assertiveness (BFASea) subscales. Neuroticism is composed of the withdrawn (BFASnw) and emotional volatility (BFASnv) subscales. Each subscale consists of 10 items for a total of 40 items.

### Analysis

Supervised machine learning algorithms are used to solve prediction problems where data is labeled (a dependent variable is specified). The full set of observations is split into training and test datasets, and the algorithm uses labels in the training set to improve accuracy while balancing generalizability to the test set. Random forest (RF) is a supervised classifier composed of an ensemble of decision trees; each of which is grown on a bootstrapped sample with a randomly selected subset of predictors, where results are aggregated by majority voting (76). In our pre-registration, we specified use of regularized regression methods, which can be used for continuous outcomes. However, RF is widely used for classification due to its robustness against skewed distributions, outliers, and data transformations (77). It has been shown to perform well in previous depression studies predicting treatment outcomes on large datasets (52).

Model performance was assessed using the following metrics. “Accuracy” refers to the proportion of cases correctly classified across all classes. “Sensitivity” refers to the proportion of cases within a specified class that were correctly classified (e.g.: the proportion of ARD observations that were predicted to be ARD by the model). “Specificity” refers to the proportion of cases not within a specified class that were correctly classified (e.g.: ARD specificity refers to the proportion of MDD and HC cases not classified as ARD by the model). As we are interested in the generalizability of models to new data, hypothesis testing was employed to assess whether test set prediction accuracy was significantly different from chance using the “no-information rate”, which is the prevalence of the largest class (78). Out-of-bag (OOB) accuracy was reported for each model, which is defined as 1 minus the average error of all predictions made using the training observations not within the bootstrapped sample. Sensitivity and specificity for training and test sets are reported for all models.

RF classifiers were implemented using the “RandomForest” (79) and “caret” (78) packages for the statistical software R (version 4.2.2) (80). Data were first divided using a pseudorandom 70/30 train/test split maintaining similar proportions of group sizes in each set (n_train_ = 276, n_test_ = 119). To avoid leakage, missing data for the train and test sets were imputed separately using predictive mean matching with the “Multiple Imputation by Chained Equations” (mice) (81) package for R. 133 observations contained at least 1 item missing in the predictor set, however total proportion of missingness in the data was low, at.4%. The variables with the highest percent missing were BFAS item 39 (2.02%), BFAS item 33 (1.77%), and BFAS item 34 (1.77%).

A multiclass target variable (all three groups) and two binarized target variables (ARD vs. non-ARD and ARD vs. MDD) were used as labels for separate models. For the first binary model, data were dummy-coded to compare ARD vs. both non-ARD groups. This model was used to generate a subset of items for distinguishing ARD from non-ARD in the general population. The other binary model was built using only the 2 depression groups. It was used to generate another subset of items for distinguishing ARD from MDD in patients with depression. Thus, the variables retained from this selection process were hypothesized to have the greatest discriminability for ARD specifically.

We defined “full models” as classifiers that included all items from the validated scales except PHQ-9, where item means were computed within each subscale (*p* = 24; where *p* is the number of predictors). Feature selection was applied separately to each model using the “VarSelRF” (82, 83) package for R, with initial number of trees = 5000 and number of trees for additional forests = 2000 (default suggested values). The algorithm uses backward elimination to drop a portion (.2) of the least important variables from the previous iteration. Using a similar process to Kautzky et al. (52), we repeated the feature selection procedure with random seeds of 1 to 500. Only those predictors retained in ≥ 80% of the results were used in “small models”.

The hyperparameter “m_try_” represents the number of predictors to be randomly sampled for each split. It is set by the experimenter and can be tuned to optimize model accuracy. We used grid search to tune m_try_ separately from feature selection with values ranging from 1 to *p*-1 using 10-fold cross-validation with 3 repeats of 500 trees each. Small models were trained and tuned separately using this method for each target variable.

Lastly, we used unsupervised methods (factor analysis and cluster analysis) to explore empirical patterns at the item level with only items from the subscales driving highest RF model accuracy. This study benefitted from empirically driven analysis due to the exploratory nature of using a novel combination of validated self-report subscales. We first conducted an exploratory factor analysis to assess if further dimension reduction would be plausible. The number of factors to extract was determined using parallel analysis (84). Factor analysis was carried out using the “psych” package for R (85) using an oblique “oblimin” rotation for factor extraction and a minimum item loading cutoff of.3. Next, *k*-means was used to explore empirical groupings of individuals (86). *K*-means is a method of clustering observations into an experimenter defined number of clusters *k*. This analysis was carried out using the “kmeans” function in the “stats” package for R, which is part of the R base code (80). *K* was determined by optimizing for within cluster sum-of-squares (WSS) using the “factoextra” package for R (87) and the “NbClust” package (88), which provides 30 indices for determining the number of clusters to use and proposes the best cluster number by majority vote. From this function, the majority of indices proposed 2 to 4 clusters. The 2-cluster solution was deemed trivial as one cluster was composed of individuals with fewer depression symptoms and the other composed of individuals with more severe depression. 3 and 4 clusters were computed for analysis and discussion.

### Data exclusion

3 subjects were excluded for failed attention checks. An additional 2 subjects were excluded due to missing multiple items comprising 1 or more subscales (predictor variable) to be used for learning, and 1 subject was excluded based on missing the target group variable. The resulting dataset comprised 393 subjects [49.0% female, mean (SD) age = 34.6(11.0)].

## Results

393 observations were included in the analysis, of which 41.7% were self-identified individuals with ARD. Unimputed PHQ-9 depression score for the full dataset significantly differed across groups (F = 24.58, *p* <.001), with post-hoc Tukey-corrected comparisons revealing significant differences between ARD vs. MDD (M_diff(ARD – MDD)_ = .32, *adjusted-p* <.001), ARD vs. HC (M_diff(ARD – HC)_ = .53, *adjusted-p* <.001) and MDD vs. HC (M_diff(MDD – HC)_ = .21, *adjusted-p* = .030; see **Supplementary Figure S1** for distribution of PHQ-9 score across groups).

78.1% of the MDD group and 67.7% of the ARD group reported taking an SSRI or SNRI for greater than 4 weeks at the time of this study. Of these individuals, 52.7% in the ARD group and 53.9% in the MDD group were taking an SSRI, 14.3% in the ARD and 20.6% in the MDD medicated group were taking an SSRI with augmentation, and 24.11% in the ARD and 18.63% in the MDD medicated group reported taking an SNRI. 24 logistic regression analyses examining the interaction effect of medication with each predictor variable regressed on group were evaluated using the generalized linear models (“glm”) function in base R (80). After correcting for multiple comparisons by controlling for a false discovery rate of <.05 using the Benjamini-Hochberg adjustment (89), no interaction effects remained significant. Therefore, no predictors were removed from the analysis. Please see **Supplementary Table S2** for mean predictor scores by group and medication status as well as their BH-adjusted p-values.

### Supervised learning

#### Multiclass target variable

The first RF model predicted group membership using the full variable set for all groups and the specified 70/30 train/test split resulting in 276 training observations with 116 events of interest (for accuracy metrics see **Table 3**). In this multiclass model, the test set accuracy (.54) was significantly higher than the no-information rate of.42 (*p* = .004). The model had the highest sensitivity for the ARD group (.71) and the highest specificity for the HC group (.86). Test sensitivity was similar to training sensitivity for all groups.

**Table 3:** Multiclass full vs. small model metrics.

		Full Model	Small Model	3-P Model	6-P Model
Train	Optimal m_try_	5	1	1	1
	OOB Accuracy	.57	.59	.51	.52
	HC sensitivity	.54	.59	.37	.34
	HC specificity	.89	.89	.83	.87
	MDD sensitivity	.42	.48	.49	.44
	MDD specificity	.77	.77	.72	.72
	ARD sensitivity	.70	.67	.61	.68
	ARD specificity	.68	.69	.69	.65
Test	Accuracy	.54**	.42	.47	.53*
	95% CI	(.45,.63)	(.33,.52)	(.38,.57)	(.43,.62)
	HC sensitivity	.45	.33	.27	.47
	HC specificity	.86	.80	.81	.85
	MDD sensitivity	.38	.28	.36	.33
	MDD specificity	.80	.78	.76	.73
	ARD sensitivity	.71	.59	.69	.71
	ARD specificity	.62	.52	.62	.68

*p <.05.

**p <.01.The full model was specified on all 24 predictors. Small model specification used only the feature selected predictors trained on the multiclass variable. 3-P and 6-P models were specified using feature selection on the binarized target variables. The no-information rate of the test set was.42.

Next, feature selection was implemented, and 5 variables retained for small model classification. The variables meeting criteria were: DASSa, MASQaa, IDASdy, MEIme, and MEIpe. These variables describe anxiety, dysphoria, as well as mental and physical energy. The ARD group had higher mean DASSa scores than the MDD group (M_diff(ARD – MDD)_ = .33) and a greater difference than the MDD vs. HC groups (M_diff(MDD – HC)_ = .01) groups. The ARD and MDD groups had a greater difference in MASQaa score (M_diff(ARD – MDD)_ = .42) than between MDD and HC groups (M_diff(MDD – HC)_ = .11). The ARD group had a higher IDASdy mean score and a greater difference in score with the MDD group (M_diff(ARD – MDD)_ = .44) than the MDD and HC groups (M_diff(ARD – MDD)_ = .35). MEIme (M_diff(ARD – MDD)_ = -.97; M_diff(MDD – HC)_ = -1.22) and MEIpe (M_diff(ARD – MDD)_ = .16; M_diff(MDD – HC)_ = -1.80) were both substantially greater in the HC group than the two depression groups.

A small model was subsequently fit using 10-fold cross validation to tune m_try_ on the training set observations with only the subset of predictors found using feature selection. Accuracy slightly improved in the training data for HC (sensitivity = .59) and MDD (.48) but decreased in test (HC sensitivity = .33; MDD sensitivity = .28; see **Table 3**); furthermore, no improvements were seen in ARD train or test sensitivity over the full model. Therefore, this small model variable set was rejected as a candidate for prediction of ARD.

#### Binarized target variables

##### ARD vs. non-ARD

This model also used 276 training observations with 116 events of interest. OOB accuracy for the full predictor set (.71) was similar to test accuracy (.65). Sensitivity was slightly higher in the test set for predicting ARD instances (.58) than the train set (.55). Full accuracy metrics for the binary target variables are reported in **Table 4**.

**Table 4:** Binarized target variables’ full vs. small model metrics.

		ARD vs. non-ARD			ARD vs. MDD		
		Full Model	Small Model	3-P Model	Full Model	2-P Model	3-P Model
Train	Optimal m_try_	3	1	1	2	1	1
	OOB Accuracy	.71	.70	.67	.69	.68	.67
	ARD Sensitivity	.55	.59	.57	.79	.77	.74
	ARD Specificity	.83	.78	.75	.57	.57	.59
Test	Test Accuracy	.65	.70**	.64	.66*	.61	.67*
	95% CI	(.56,.74)	(.61,.78)	(.55,.73)	(.55,.76)	(.50,.72)	(.56,.77)
	ARD Sensitivity	.57	.63	.59	.86	.71	.82
	ARD Specificity	.71	.75	.68	.41	.49	.49

*p <.05.

**p <.01. The m_try_ hyperparameter was optimized on accuracy, which is the proportion of correct categorizations. OOB accuracy represents the proportion of correct cases not within each bootstrapped sample for all classes. Test accuracy represents the proportion of correctly classified cases in the test set for all classes. Sensitivity represents correctly classified cases of the selected class. Specificity represents correctly classified cases not of the selected class. Full model specification included all 24 predictor variables. Small model specification included only the variables meeting selection criteria when trained on the corresponding target variable’s training set. 3-P and 2-P models were specified using the feature selection procedure with the ARD vs. MDD target variable.

Feature selection of the ARD vs. non-ARD target variable retained 5 variables. All the DASS subscales were retained in addition to IDASdy and MEIme. The difference in mean score for these subscales (except MEIme, which had similarly large differences between all groups) were greater between ARD vs. MDD than MDD vs. HC (DASSd: M_diff(ARD – MDD)_ = .40; M_diff(MDD – HC)_ = .22; DASSs: M_diff(ARD – MDD)_ = .34; M_diff(MDD – HC)_ = .07). Fitting a 10-fold cross validated model for m_try_ on the small set of variables resulted in improved overall test accuracy and a significant p-value for the hypothesis test of accuracy evaluated against the no-information rate of.58 (accuracy = .70, *p* = .005). Sensitivity (.63) and specificity (.75) for ARD improved moderately in the small model.

##### ARD vs. MDD

This model was specified on 205 training observations and 116 events of interest. OOB accuracy on the full predictor set was.69 and.66 for test accuracy. Sensitivity for ARD cases in the cross-validated train data reached.79, however the model did not generalize as well (ARD test sensitivity = .65). Feature selection resulted in only 2 variables retained under pre-defined criteria: DASSd and MEIsm. The MEIsm subscale was surprisingly greater in the ARD group than the MDD group (M_diff(ARD – MDD)_ = .21). Test accuracy (.61) and ARD sensitivity (.71) were somewhat improved in this model. In the interest of finding a set of variables with improved discriminability for ARD, we also computed a 3-predictor (3-P) model including the next most frequent variable selected (48% of random seed iterations): DASSa. This model demonstrated a significantly greater test accuracy over the no-information rate of.56 (accuracy = .67, *p* = .020) with a test sensitivity for ARD of.82 and specificity of.49. Both the 2-P and 3-P small models had the same specificity (.49) for ARD; thus the 3-P model substantially increased accuracy of classifying ARD cases, but not at the expense of MDD accuracy.

#### Generalization of binarized feature selection on multiclass target variable

The set of predictors resulting in the greatest sensitivity to ARD included: DASSd, DASSa, and MEIsm. This set was used to generate predictions for the other target variables. Using the 3-predictor (3-P) model to train on the multiclass target variable resulted in lower sensitivities for HC (.27) and MDD (.36) in the test set, but slightly improved sensitivity for ARD (.69). However, sensitivity did not improve in the ARD vs. non-ARD target variable using this test set.

We also tested a combined 6-predictor (6-P) model using predictors retained from both binarized target variables’ feature selection processes on the multiclass variable. This resulted in a small increase in overall test accuracy, which was significant over the no-information rate (*p* = .010). ARD test sensitivity (.71) was improved over the other multiclass models, however MDD (.33) and HC (.47) sensitivity remained low. Full results are reported in **Table 3**.

### Unsupervised learning

In the following, only individual items from the 6-P model subscales were analyzed: DASSd, DASSa, DASSs, MEIme, MEIsm, and IDASdy. Only a very small percentage of observations were missing from this subset (.03%). Therefore, to increase clarity of data interpretation without a large risk of introducing bias, predictive mean matching was used to impute missing data based only on this subset of items using the “mice” package (81).

#### Exploratory factor analysis

We used exploratory factor analysis to confirm whether the factor structure at the item level would be retained when combining items from multiple validated scales. Parallel analysis suggested 6 factors in the item-level data. The 6-factor solution item loadings are reported in **Supplementary Table S3**. Most items grouped into their theoretically proposed subscales. We interpret the factors in their order of extraction. The first factor was composed of anhedonia items (mostly DASSd: “*I couldn’t seem to experience any positive feeling at all*”). The second factor was composed mostly of somatic anxiety items (DASSa: “*I had a feeling of faintness*”) and some DASSs items (“*I was in a state of nervous tension*”). Factor 3 was composed of cognitive function items (MEIme: “*During the past 4 weeks, how often did you have problems concentrating?*”). Factor 4 was composed solely of DASSs items related to distress (“*I found myself getting upset by quite trivial things*”). Factor 5 was composed almost solely of MEIsm items (“*During the past 4 weeks, to what extent were you interested in talking with others?*”). Factor 6 was composed of 4 IDAS dysphoria items related to self-worth and guilt (“*I felt inadequate*”). However, other items from the IDASdy subscale loaded onto the first 3 factors. Only one item did not have a loading >.3 onto any factors (MEIsm: “*During the past 4 weeks, how often did you avoid social conversations with others?*”).

#### Cluster analysis

**Figure 1:**
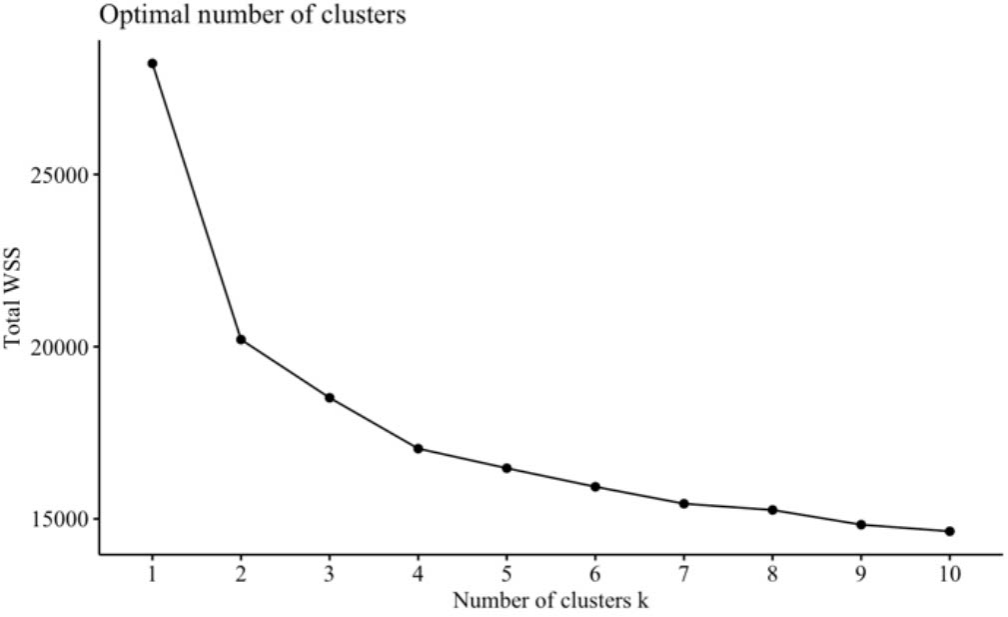
Number of clusters. Total WSS is plotted by number of clusters. Beyond *k* = 4, the WSS incrementally decreases at a decreasing rate. Therefore, a 4-cluster solution was chosen to prevent unnecessary complexity and inaccuracies to data modeling and interpretation.

**Figure 2:**
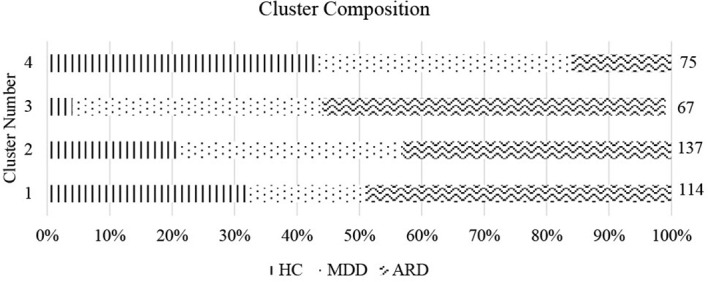
Cluster composition by group. Cluster number and proportion by group (% of total cluster) are shown for *k* = 4. Size of each cluster is indicated on the right vertical axis.

We performed cluster analysis of individuals using the subset of individual items within the 6-P model using 4 clusters, determined from optimizing for WSS. A cluster by WSS graph is presented in **Figure 1**. Due to our sample having 3 diagnostic groups, we also computed a 3-cluster solution. In the 3-cluster solution: cluster 1 was composed mostly of individuals from the MDD and ARD groups. Cluster 2 consisted mostly of the ARD group, and cluster 3 consisted mostly of HC and MDD groups. In the 4-cluster solution: cluster 1 consisted of mostly HC and ARD individuals. Clusters 2 and 3 consisted of mostly the MDD and ARD groups, while cluster 4 was mostly the HC and MDD groups. Cluster by group frequencies are shown in **Figure 2** for the *k* = 4 solution.

To characterize differences in symptom profiles across clusters, we computed means of the standardized item scores for each of the 6 factor-analyzed dimensions. Results are summarized in **Table 5**.

Table 5Factor means by cluster. Standardized item score factor mean and SDs by cluster are reported for *k* = 3 (top) and *k* = 4 (bottom).

**Table:** 

6-Factor Standardized Item Scores by Cluster: *k* = 3					
Factor	1, N = 125*^1^*	2, N = 138*^1^*	3, N = 130*^1^*	p-value*^2^*	q-value*^3^*
Anhedonia	.15 (.64)	.65 (.47)	-.84 (.40)	<.001	<.001
Anxiety	-.18 (.38)	.84 (.34)	-.72 (.32)	<.001	<.001
Cognitive	-.09 (.31)	-.22 (.28)	.32 (.28)	<.001	<.001
Distress	.00 (.53)	.73 (.42)	-.77 (.44)	<.001	<.001
Motivation	-.44 (.43)	.24 (.72)	.16 (.60)	<.001	<.001
Dysphoria	.33 (.73)	.40 (.64)	-.74 (.65)	<.001	<.001

^1^ Mean (SD).

^2^ Kruskal-Wallis rank sum test.

^3^ False discovery rate correction for multiple testing.

**Table:** 

6-Factor Standardized Item Scores by Cluster: *k* = 4						
Factor	1, N = 114*^1^*	2, N = 137*^1^*	3, N = 67*^1^*	4, N = 75*^1^*	p-value*^2^*	q-value*^3^*
Anhedonia	.48 (.35)	-.32 (.43)	.98 (.54)	-1.04 (.36)	<.001	<.001
Anxiety	.83 (.34)	-.35 (.36)	.30 (.56)	-.90 (.18)	<.001	<.001
Cognitive	-.19 (.28)	.04 (.32)	-.22 (.30)	.41 (.25)	<.001	<.001
Distress	.69 (.36)	-.27 (.46)	.48 (.64)	-.99 (.37)	<.001	<.001
Motivation	.52 (.48)	-.21 (.46)	-.75 (.38)	.26 (.65)	<.001	<.001
Dysphoria	.19 (.54)	-.11 (.61)	1.08 (.46)	-1.06 (.52)	<.001	<.001

^1^ Mean (SD).

^2^ Kruskal-Wallis rank sum test.

^3^ False discovery rate correction for multiple testing. Positive means denote above-average levels and negative means denote below-average levels of each factor. The anhedonia factor describes low pleasure and interest and was composed mostly of DASSd items. The anxiety factor was composed mostly of DASSa and DASSs items and describes somatic symptoms of nervousness. The cognitive factor describes cognitive functioning (i.e. focus, memory and decision-making), and was composed mostly of MEIme items. The distress factor was composed of DASSs items related to emotional upset. The motivation factor was composed of MEIsm items related to social and recreational motivation, and the dysphoria factor was composed of IDASdy items related to guilt and self-worth.

##### 3-cluster solution

The first cluster in the *k* = 3 solution can be interpreted as capturing the similarities between the 2 depression groups (n_HC/MDD/ARD_ = 15/47/63). This cluster had the lowest motivation and above average levels of anhedonia and dysphoria as well as below average anxiety. The second cluster (n_HC/MDD/ARD_ = 36/30/72) was composed of mostly the ARD group. This cluster displayed the highest anxiety, anhedonia, distress, and dysphoria coupled with the lowest cognitive functioning. Interestingly, this cluster also displayed above average social motivation. The 3rd cluster (n_HC/MDD/ARD_ = 49/52/29) was composed mostly of the HC group, and displayed low anhedonia, anxiety, distress, and dysphoria along with above average cognitive functioning and motivation.

##### 4-cluster solution

**Figure 3:**
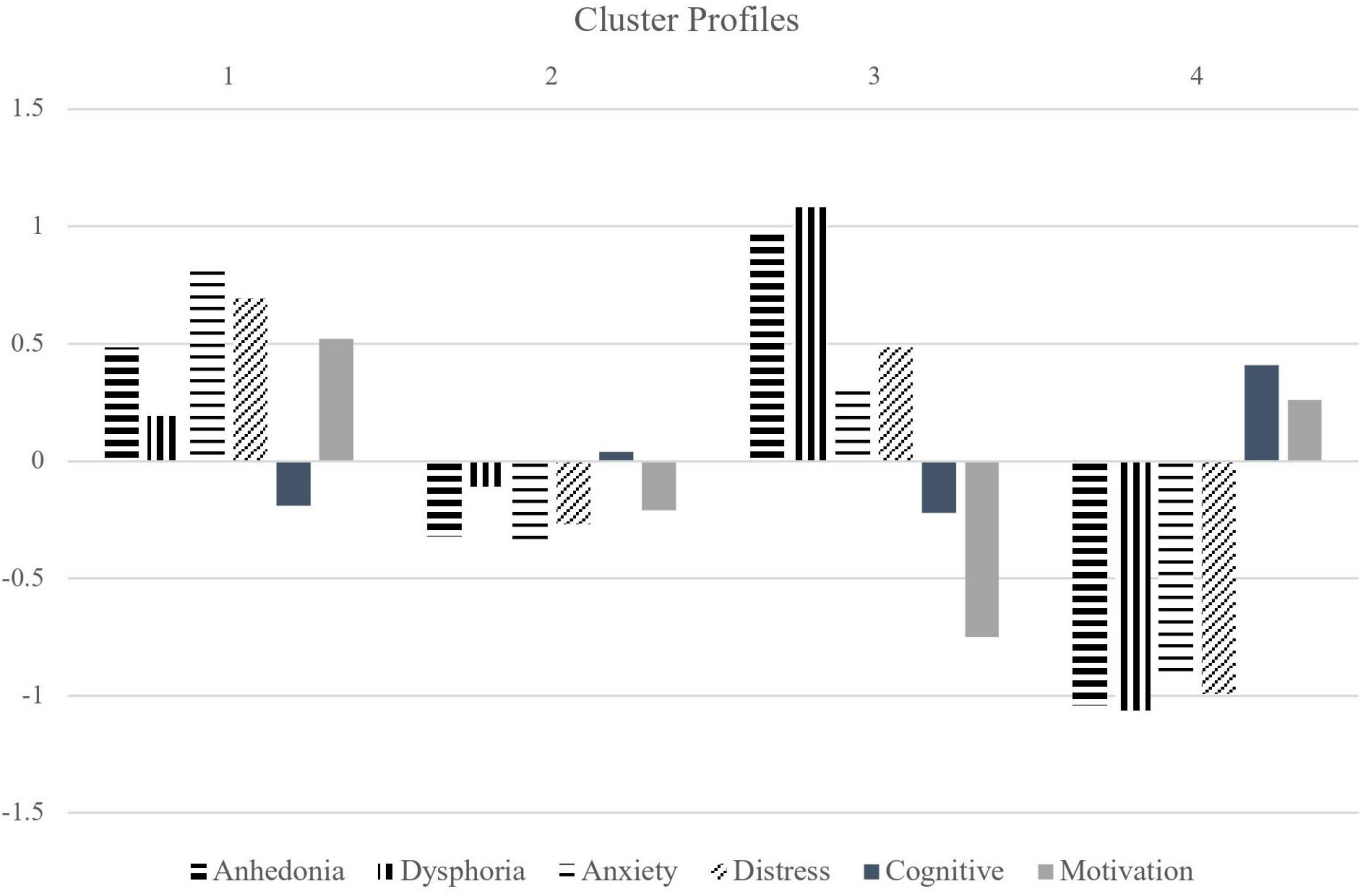
Anhedonia factor cluster profiles. (from left to right) The first 4 factors are negatively valenced. The last 2 factors are positively valenced (cognitive functioning and motivation) and depicted using solid bars.

The first cluster (n_HC/MDD/ARD_ = 36/22/56) in the k = 4 solution was composed mostly of commonalities between ARD and HC groups. This cluster was characterized by the highest anxiety of all clusters, along with above average anhedonia, distress, and dysphoria and below average mental functioning. Similar to cluster 2 in the 3-cluster model, this cluster also had high motivation. Cluster 2 (n_HC/MDD/ARD_ = 29/49/59) was composed mostly of commonalities between MDD and ARD groups, and displayed low anhedonia, anxiety, distress and dysphoria along with low motivation. Cluster 3 (n_HC/MDD/ARD_ = 3/27/37) was composed of mostly ARD individuals and displayed the highest levels of anhedonia and the lowest levels of motivation and cognitive functioning. This group also had the highest levels of dysphoria and above average distress and anxiety. Cluster 4 (n_HC/MDD/ARD_ = 32/31/12) represented similarities between HC and MDD groups, and displayed the lowest levels of anhedonia, anxiety, distress, and dysphoria. This group also had above average motivation and cognitive functioning. See **Figure 3** for graphical depiction of cluster profiles.

## Discussion

Random forest classification models built using anhedonia and other internalizing predictors reached accuracy levels ranging from.42-.71. Sensitivity for ARD ranged from.55-.86 and specificity ranged from.49 -.83 (proportion of ARD individuals in the total sample = .42 and in the depression only groups = .56). Model performance based on anhedonia and related predictors was comparable to results reported from other studies using comprehensive sets of demographic, socioeconomic and clinical predictors from cohort depression databases (44, 49, 52, 90, 91). Several models including the full multiclass comparison reached test accuracy levels significantly above chance. The small model with best overall accuracy and sensitivity for ARD contained 6 predictors and factor analyzed into 6 symptom dimensions at the item level. Cluster analyses revealed 3-4 empirical groupings varying in affective and cognitive disturbance along these 6 dimensions.

Optimal m_try_ varied between 1-5 and was generally smaller for each cross-validated model than the recommended rule of thumb (m_try_ = √predictors; for a plot of accuracy by m_try_ values for the multiclass variable full model, see **Supplementary Figure S4**). Larger values of this hyperparameter generate more optimized forests, as the best predictor can more often be chosen for each split. However, this can also lead to overfitting. Smaller m_try_ values lead to a weaker but more diverse forest as only a few predictors are tested at a time, and models can generalize better when making predictions for test set observations. All small models performed best with m_try_ = 1.

Feature selection of the binarized comparisons (the 6-P model) resulted in a set of variables with greater discriminability for ARD. The 6-P model performed better in test sensitivity/specificity for ARD in the multiclass comparison than the set of selected variables specified using the multiclass target variable itself. These variables encompassed the measurement of low pleasure (“*I couldn’t seem to experience any positive feeling at all*”), motivation (“*During the past 4 weeks, how often did you engage in recreational activities or hobbies?*”), cognitive function (“*During the past 4 weeks, how often did you have trouble making minor decisions?*”), stress (“*I found myself getting upset rather easily*”), and anxiety (“*I felt that I was using a lot of nervous energy*”), whereas the set of items specified using the multiclass target contained predictors related more to somatic anxiety and physical energy. Therefore, somatic symptoms may be more important in identifying any depression whereas anhedonia and cognitive symptoms may be more important for specifically identifying individuals with ARD.

Cognitive function as measured by MEIme and cognitive distress as measured by IDASdy displayed similarly large differences across ARD vs. MDD and MDD vs. HC groups. Cognitive impairment in the areas of attention, executive function, and memory are considered core markers of major depression and have been found to persist even after depression remission (92, 93). Dysphoria, being the opposite of euphoria, was composed of items representing thoughts of worthlessness, hopelessness and guilt. It may be related to emotion dysregulation, and has been associated with depressive episodes and cognitive impairment (94, 95). These findings suggest that individuals with ARD are much more cognitively impaired than non-depressed individuals and are more likely to suffer from disordered thinking and worry. Untreated, this may lead to greater risk for recurrent depressive episodes.

The anxiety and stress scores displayed greater differences between ARD vs. MDD than MDD vs. HC. This aligns with previous studies finding comorbid anxiety to be a clinical predictor of treatment resistance (90, 96, 97). The importance of anxiety measurement is highlighted again when distinguishing solely between ARD and MDD groups. In this comparison, the full model performed significantly above chance whereas the 2-P model (using only items representing depressed mood and social motivation) did not. However, the 3-P model performed comparably to the full model in terms of test sensitivity and specificity for ARD. Adding an anxiety subscale improved predictive sensitivity for ARD without sacrificing specificity. According to these results, high negative emotionality is equally important as anhedonia (low positive emotionality) for ARD discriminability.

Reported physical energy (MEIpe: “*During the past 4 weeks, how much of the time did you feel physically tired during the day?*”) was much higher in healthy controls; HC vs. MDD had a substantially greater difference in mean score than MDD vs. ARD. Similarly, the MEIsm mean score was much greater in HC than the 2 depression groups. This subscale captured more than just social motivation; it also contained items related to interest in recreational activities and projects. Thus, a physical component of anhedonia may be more important for distinguishing individuals with and without depression. Physical activity is a well-established strategy for management of depression symptoms, and frequently recommended for prevention of depression (98–100). Furthermore, exercise has been found to be beneficial in the treatment of anxiety, and in a non-clinically depressed population for improvement of depression symptoms (101, 102). Individuals with ARD report low physical energy coupled with high anxiety. Therefore, these individuals may stand to gain the most from physical activities, but paradoxically may have the most trouble implementing a regular exercise protocol.

This study demonstrates the viability of using a limited set of self-report predictors relating to one broad symptom dimension for classification of antidepressant response. The range of sensitivity achieved for ARD in this study (.55-.86) was comparable to other naturalistic machine learning studies using a more diverse set of sociodemographic, diagnostic and medical history, genetic and self-report clinical predictors (.55 -.82) (49–52). Such comprehensive information can be impractical to gather for every patient and not readily available in real-life clinical practice. Additionally, unlike the previously cited studies leveraging national depression databases, we included a control sample of individuals with no reported depression diagnosis or treatment history. Therefore, this study was not limited in scope to binary comparisons of response vs. non-response and achieved classification accuracy at levels significantly above chance with a multiclass comparison. Furthermore, the feature selection process elucidated the existence of distinct differences in anhedonia profiles within a depression group and between individuals with and without depression. This study contributes to our understanding of the nature of SSRI/SNRI treatment resistance and highlights a novel pathway for clinical application.

Few prior studies using machine learning to predict treatment outcomes have examined the meaning of variable and model selection results for improving our understanding of clinical phenotypes. In this study we used unsupervised methods to reveal data-driven insights on the symptom profile(s) of ARD. Exploratory factor analysis mostly retained the 6-P scale dimensions, except IDASdy. Dysphoria is a multifaceted component of depression and represents a general dissatisfaction and unease toward life. The IDASdy subscale was composed of items capturing distress, worry, low self-worth, hopelessness, and guilt (67). Therefore, several items were split among the other dimensions of anxiety, anhedonia, and cognitive impairment. However, 4 items loaded together to form a low self-worth and hopelessness factor (“I felt discouraged about things”; “I blamed myself for things”), which retained the name dysphoria.

These dimensions were then examined across empirically determined data clusters. The 4-cluster solution found 2 large (capturing around 2/3 of the total cases) and 2 smaller clusters (approx. 1/3 of the total cases combined) such that ARD was evenly distributed across the 2 large clusters and dominated one of the smaller clusters. The first large cluster resembled the symptom profile of an anxious depression subtype, with greater reported levels of anxiety and distress than anhedonia and dysphoria. This cluster also displayed above average levels of motivation, presumably because motivation can be derived from anxious avoidance of aversive events or end states (41, 103, 104). This cluster was composed of proportionally more HC and ARD as well as fewer MDD individuals than the second large cluster. The second large cluster was composed of a low-disturbance profile characterized by slightly below average anxiety, distress, anhedonia, motivation, and dysphoria. The symptom profile of this cluster suggests a successfully treated group of participants. The third cluster was a small group consisting of mostly ARD participants. The symptom profile of this cluster was characterized by exceedingly high anhedonia, dysphoria, low motivation, plus moderate cognitive impairment, and above average anxiety and distress. However, unlike cluster 1, the anhedonia and dysphoria in this cluster was greater than the anxiety and distress symptom dimensions, while motivation was much more impaired. Cluster 4 was composed mainly of the HC and MDD groups. This cluster scored below average on the negatively valenced symptom dimensions (anhedonia, dysphoria, anxiety, and distress) and above average on the positively valenced dimensions (cognitive function and motivation). Therefore, we found 4 clusters of participants based on internalizing symptom profiles, loosely resembling the subtypes (cluster 1) anxious-depression, (cluster 2) low-disturbance/treated, (cluster 3) anhedonic, and (cluster 4) non-depressed.

These findings suggest the presence of symptom heterogeneity even in just individuals with ARD, varying along dimensions of anxiety, anhedonia, and cognitive disturbance. Some of the variation in profiles may have been driven by the presence of antidepressant medication. In both the 3- and 4-cluster solution, a similar low anxiety depression profile was present and contained the greatest proportion of the non-resistant MDD group. This may reflect the robust anxiolytic effects of SSRI/SNRI medication; indeed, several SSRIs are indicated for treatment of anxiety disorders, post-traumatic stress disorder, and obsessive-compulsive disorder (105–107). Additionally, emotional blunting is a commonly reported side-effect of this type of medication (108–110).

Previous evidence has suggested a mechanistic difference underlying the interest and the pleasure facets of reward processing (19, 32, 34). The TEPSa, BFASee, and BASd subscales all reflect the interest component of anhedonia related to function of the dopaminergic reward circuit. To be in line with recent evidence for the successful treatment of anhedonia using neural stimulation of reward circuit regions, we would expect much larger differences between these subscales for the ARD vs. MDD groups (111–113). However, to the extent that this can be captured in self-report data, we did not find differences in anticipatory anhedonia and apathy between ARD and MDD to be robust at the level of granularity posited. We did, however, find the importance of a broad internalizing dimension composed of affective and cognitive disturbances in contributing to prediction of a treatment-resistant phenotype.

Lastly, it is important to note that the majority of individuals with depression in both the MDD and ARD groups were taking some form of serotonergic medication, and SSRIs were the most common treatment even for individuals who self-identified as ARD. This demonstrates the pervasiveness of serotonergic medication use in depression treatment, even as its high non-response rate is widely accepted (58). The dominant narrative of the monoamine hypothesis of depression was a major limiting factor for identifying new treatment mechanisms (114). Additionally, current perspectives on alternative treatments are that they carry greater risks; for example, deep brain stimulation and electroconvulsive therapy are well-established for treating non-responsive depression (16, 115, 116), but use invasive surgical techniques. Esketamine and other pharmacotherapies are nascent treatments with promise for efficacy, but some researchers still question long-term safety and tolerability (117). However, there is increasing acknowledgment of the heterogeneity in major depression, and many recognize the need for diversification and individualization of treatment protocols (118).

### Limitations

Several limitations were present due to the cross-sectional nature of this study. First, treatment at time of study adds a complex confound to the interpretation of these results, as people were prescribed varying doses of medications from different antidepressant classes and may augment with differing classes of medications or alternative therapies. However, the overwhelming majority of participants who were on medication listed an antidepressant within the SSRI/SNRI pharmacological classes, and these medications are ineffective at reducing anhedonia (7). Similarly, chronic use of recreational substances and drugs of abuse may contribute another confound. The survey items generally measured across multiple days or weeks and trait-level effects, which may somewhat reduce bias from acute substance use. Yet these are two sources of bias that must be considered when interpreting findings.

A second limitation arises from the online case-control study design and self-diagnosed group labels. Reliance on online self-report modality is a simple way to streamline large-scale data collection from a broad geographical area with a greater level of confidentiality for participants. However, it can impact internal validity due to the inability to verify accuracy of self-reporting and standardize phenomenological measurement. Furthermore, there may be impacts to external validity and generalizability to patient populations who do not participate in online research platforms. Therefore, more extensive research is needed to validate these findings through both verification of patient records and in-person data collection across several geographic locations.

Clinicians frequently use subjective reports of symptom improvement when making treatment modifications. However, in this study the measure of improvement from medication was not standardized and must be interpreted with caution. We were not able to measure pre to post change in depression, as we only captured respondents at one time point. Because of these limitations, we are not able to draw conclusions about pre-treatment anhedonia profile. In our data, there were large ranges for depression severity in each self-identified group; distributions can be seen in **Supplementary Figure S1**. Despite efforts to limit the presence of depression in the HC group by pre-screening based on PHQ-2, a substantial portion of this group still averaged a moderate score on the PHQ-9 and reported presence of other internalizing symptoms such as anxiety on the other measures. Therefore, the HC group had somatic and cognitive symptoms (as only affective symptoms are assessed by PHQ-2). This suggests a substantial portion of the population may express anxious depression symptoms while not considering themselves depressed or not seeking a diagnosis. This may be due to (1) a component of alexithymia that may be present in mood disorders, or (2) lack of general knowledge around the heterogeneity of depression criteria and failure to recognize the somatic and cognitive impairments that define both depression and anxiety. In addition, the MDD group appeared to have a bimodal distribution on PHQ-9 scores, and some also reported various internalizing symptoms based on the cluster analysis. Therefore, some individuals may have self-identified as responding to medication while contending with residual symptoms, due to the heterogeneous nature of depression symptom dimensions they may have felt improvement in some domains while remaining static in others. This demonstrates a limitation of self-identified diagnosis; significant variability exists in these groups.

Third, Random Forests is limited in its use outside the bounds of this dataset because it cannot extrapolate predictions for new values. Therefore, it is bound by the largest and smallest values of predictors in the training set. Additionally, sample size differences across classes can sometimes contribute to variation in sensitivity and specificity for the models, with sensitivity skewed toward the larger class. In the multiclass models, ARD sensitivity was generally greater than the other 2 groups. The sample size of the ARD group was slightly greater than the HC (1.64:1) and MDD (1.27:1) groups. The binarized models performed better in accuracy than the multiclass, with the ARD vs. non-ARD model performing better for classifying non-ARD cases and the ARD vs. MDD model better at classifying ARD cases. In the first binarized model, the combined sample size for the HC and MDD groups was slightly larger than the ARD group (1.4:1), while the sample size in the second binarized model was slightly larger in the ARD group (.8:1). Data balancing is sometimes used with RF classifiers to prevent this issue, and can be performed by a combination of under-sampling the majority class and over-sampling the minority class (119). However, this can introduce some bias in the data; therefore, it is commonly used for extremely unbalanced data (minority class prevalence < 10%). The results are thus better interpreted by comparing full to small model accuracy across all groups. To address these limitations, results should be replicated in a sample of pre-treatment individuals with depression who are followed longitudinally from pre- to post-treatment and assessed by a clinician for depression improvement.

## Conclusions

These findings highlight the efficacy of using a limited set of self-reported anhedonia and internalizing predictors to evaluate SSRI/SNRI treatment-resistance. This case-control study attained comparable model performance to prior naturalistic cohort studies exploiting a broader range of sociodemographic and clinical predictors without using a non-depressed control group. Specific components of internalizing symptomatology (i.e. depressed mood) were found to have greater importance for distinguishing ARD in particular, whereas other components (i.e. physical energy) were more relevant for distinguishing any presence of depression. Furthermore, an abridged set of items relating to anxiety, anhedonia, and cognitive function were found to differentiate ARD from non-ARD individuals at levels significantly above chance. This study found (1) the qualitative components of anhedonia differ when comparing across treatment response groups vs. overall presence of disorder, and (2) produced a reasonable sized set of items consisting of the DASS, MEI mental energy and social motivation subscales, and IDAS dysphoria subscale for practical clinical use. Self-report items are easy to administer, standardized, and cost friendly. To enhance usability by clinicians as a predictive tool for ARD in pre-treatment individuals, further study should aim to replicate results in a prospective cohort sample.

## Data availability statement

The original contributions presented in the study are included in the article/**Supplementary Material**, further inquiries can be directed to the corresponding author/s.

## Ethics statement

The studies involving humans were approved by University of Southern California Institutional Review Board. The studies were conducted in accordance with the local legislation and institutional requirements. The participants provided their written informed consent to participate in this study.

## Author contributions

XL: Conceptualization, Data curation, Formal analysis, Funding acquisition, Methodology, Writing – original draft. SR: Resources, Supervision, Writing – review & editing.
