# Supplementary material for: Development of a multivariate prediction model for antidepressant resistant depression using reward-related predictors
Source: Front Psychiatry. 2024 Mar 25;15:1349576. doi: 10.3389/fpsyt.2024.1349576 (PMC10999634; doi:10.3389/fpsyt.2024.1349576)
Supplement: Supplementary file 1 [file DataSheet_1.docx]

Supplementary Material

**Anhedonia subtype profiles determine serotonergic antidepressant response significantly above chance**

Xiao Liu*, Stephen J. Read

*** Correspondence:** Xiao Liu: [xliu5899@usc.edu](mailto:xliu5899@usc.edu)

**Machine Learning Methods**

Machine learning methods provide advantages in classification over traditional logistic regression in several ways: (1) logistic regression assumes a linear relationship between the predictors and the log-odds of the response variable, but many real-world relationships are nonlinear; (2) machine learning models can handle high-dimensional data and have the capacity to perform feature selection or extraction to improve generalization; (3) ensemble techniques (i.e., Random Forests, Gradient Boosting, or Stacking), where multiple models are combined to make predictions can reduce overfitting, improve generalization, and lead to better predictive performance; (4) logistic regression can be sensitive to outliers, and their presence can distort the model's performance, but machine learning algorithms like Random Forests are less affected by outliers due to their ensemble nature; (5) machine learning models can automatically capture interactions between variables and complex interdependencies, which may be missed by simple linear or logistic regression (1).

1. Bzdok D, Meyer-Lindenberg A. Machine Learning for Precision Psychiatry: Opportunities and Challenges. Biol Psychiatry Cogn Neurosci Neuroimaging. 2018 Mar 1;3(3):223–30.

# Supplementary Tables

|  | **Meds** | | **No Meds** | | ***BH adjusted p*** |
| --- | --- | --- | --- | --- | --- |
| **Predictor** | **ARD**, N = 111*^1^* | **MDD**, N = 101*^1^* | **ARD**, N = 53*^1^* | **MDD**, N = 28*^1^* | predictor * meds |
| PHQ_mean | 1.54 (.60) | 1.17 (.67) | 1.54 (.61) | 1.37 (.60) | -- |
| MEIme_mean | 5.08 (1.84) | 6.28 (2.29) | 5.22 (2.34) | 5.46 (2.02) | .244 |
| MEIpe_mean | 4.88 (2.04) | 4.58 (2.30) | 3.82 (2.19) | 3.66 (2.34) | .909 |
| MEIsm_mean | 7.94 (2.66) | 7.13 (2.96) | 5.20 (3.10) | 5.80 (3.00) | .261 |
| TEPSc_mean | 4.10 (.77) | 4.36 (.94) | 4.03 (.96) | 4.25 (.83) | .988 |
| TEPSa_mean | 3.76 (.66) | 3.88 (.75) | 3.23 (.83) | 3.56 (.71) | .630 |
| MASQaa_mean | 2.77 (.89) | 2.21 (.93) | 2.14 (.76) | 1.95 (.70) | .656 |
| MASQad_mean | 3.42 (.97) | 3.68 (.90) | 4.33 (.68) | 4.11 (.68) | .252 |
| MASQgd_mean | 3.23 (.79) | 2.71 (.95) | 3.18 (.85) | 3.03 (.98) | .319 |
| BFASnw_mean | 3.39 (.58) | 3.30 (.75) | 3.64 (.78) | 3.54 (.85) | .984 |
| BFASnv_mean | 3.15 (.60) | 2.98 (.82) | 2.94 (.94) | 3.22 (.94) | .328 |
| BFASee_mean | 2.86 (.56) | 3.07 (.81) | 2.45 (.75) | 2.81 (.86) | .965 |
| BFASea_mean | 2.97 (.64) | 2.82 (.83) | 2.81 (.95) | 2.91 (.87) | .424 |
| IDASdy_mean | 3.24 (.78) | 2.72 (.93) | 3.13 (.79) | 2.94 (.94) | .439 |
| IDASla_mean | 3.32 (.88) | 2.94 (.85) | 3.06 (.85) | 3.25 (1.01) | .228 |
| DASSd_mean | 1.60 (.68) | 1.15 (.77) | 1.58 (.84) | 1.35 (.73) | .423 |
| DASSa_mean | 1.36 (.66) | .88 (.75) | .80 (.57) | .73 (.62) | .292 |
| DASSs_mean | 1.58 (.58) | 1.09 (.70) | 1.24 (.63) | 1.28 (.61) | .096 |
| BIS_mean | 2.88 (.47) | 2.99 (.47) | 3.08 (.44) | 3.05 (.45) | .456 |
| BASd_mean | 2.69 (.59) | 2.41 (.71) | 2.22 (.63) | 2.10 (.62) | .698 |
| BASr_mean | 2.87 (.60) | 3.04 (.70) | 2.86 (.63) | 2.95 (.46) | .943 |
| BASf_mean | 2.75 (.62) | 2.49 (.66) | 2.38 (.66) | 2.47 (.68) | .216 |
| ACIPSgs_mean | 3.93 (.93) | 4.21 (1.35) | 3.36 (1.48) | 3.79 (1.26) | 1.000 |
| ACIPSis_mean | 3.98 (.80) | 4.23 (1.07) | 3.60 (1.12) | 3.78 (.99) | .906 |
| ACIPSsb_mean | 4.04 (.94) | 4.14 (1.09) | 3.58 (1.07) | 3.69 (1.12) | .983 |
| *^1^* Mean (SD) |  |  |  |  |  |

**S2.** Item means and standard deviations by depression medication status and group, as well as FDR controlled p-values.

|  | **Anh** | **Anx** | **Cog** | **Dis** | **Mot** | **Dys** |
| --- | --- | --- | --- | --- | --- | --- |
| DASSd37 | **0.83** | 0.03 | 0.02 | 0.03 | -0.02 | -0.07 |
| DASSd38 | **0.80** | 0.10 | 0.11 | 0.07 | 0.05 | -0.01 |
| DASSd21 | **0.77** | 0.06 | 0.04 | 0.11 | 0.08 | -0.05 |
| DASSd34 | **0.75** | 0.02 | -0.02 | 0.05 | 0.06 | 0.14 |
| DASSd10 | **0.73** | 0.11 | -0.02 | 0.00 | -0.07 | -0.02 |
| DASSd17 | **0.72** | 0.05 | 0.05 | 0.11 | 0.07 | 0.15 |
| DASSd26 | **0.69** | -0.10 | -0.13 | 0.09 | 0.01 | 0.14 |
| DASSd31 | **0.66** | 0.17 | -0.07 | 0.01 | -0.07 | -0.14 |
| DASSd13 | **0.65** | -0.05 | -0.13 | 0.09 | -0.02 | 0.09 |
| DASSd3 | **0.65** | 0.12 | -0.06 | 0.05 | -0.06 | -0.05 |
| DASSd24 | **0.59** | 0.17 | -0.15 | 0.01 | -0.11 | -0.03 |
| IDASdy8 | **0.59** | -0.12 | -0.23 | 0.02 | -0.06 | 0.20 |
| DASSd16 | **0.57** | 0.18 | -0.14 | 0.05 | -0.13 | -0.10 |
| IDASdy2 | **0.40** | 0.15 | -0.22 | 0.02 | -0.12 | 0.04 |
| DASSa15 | 0.13 | **0.69** | -0.08 | -0.05 | 0.12 | -0.15 |
| DASSa7 | 0.11 | **0.69** | -0.05 | 0.01 | 0.00 | -0.05 |
| DASSa20 | 0.10 | **0.67** | -0.02 | 0.09 | 0.02 | 0.06 |
| DASSs33 | 0.02 | **0.67** | 0.00 | 0.12 | -0.02 | 0.23 |
| DASSa40 | 0.02 | **0.66** | 0.03 | 0.15 | -0.03 | 0.21 |
| DASSa4 | 0.07 | **0.66** | -0.03 | 0.04 | 0.10 | -0.12 |
| DASSa41 | 0.11 | **0.66** | -0.05 | 0.07 | 0.10 | -0.10 |
| DASSa25 | -0.05 | **0.65** | -0.09 | 0.05 | 0.00 | -0.05 |
| DASSa28 | 0.11 | **0.63** | -0.07 | 0.10 | -0.04 | 0.08 |
| DASSa36 | 0.18 | **0.62** | 0.04 | 0.10 | 0.06 | 0.03 |
| DASSa23 | 0.07 | **0.58** | -0.04 | 0.13 | 0.20 | -0.19 |
| DASSs12 | -0.02 | **0.51** | -0.09 | 0.08 | -0.03 | 0.33 |
| DASSa19 | 0.05 | **0.51** | 0.01 | 0.26 | 0.06 | -0.11 |
| DASSa2 | 0.02 | **0.51** | -0.22 | -0.08 | 0.04 | -0.08 |
| IDASdy57 | 0.14 | **0.50** | -0.08 | 0.13 | 0.21 | -0.18 |
| IDASdy5 | -0.06 | **0.49** | -0.22 | 0.10 | -0.01 | 0.19 |
| DASSa9 | 0.01 | **0.47** | -0.12 | 0.15 | -0.13 | 0.28 |
| DASSs22 | 0.09 | **0.43** | -0.20 | 0.08 | 0.05 | 0.07 |
| DASSs8 | 0.13 | **0.41** | -0.19 | 0.06 | -0.08 | 0.25 |
| DASSa30 | 0.16 | **0.40** | -0.06 | 0.24 | 0.05 | 0.10 |
| MEIme9 | 0.09 | -0.09 | **0.81** | -0.05 | -0.02 | 0.02 |
| MEIme5 | -0.12 | 0.08 | **0.75** | -0.05 | -0.07 | 0.03 |
| MEIme13 | -0.05 | 0.08 | **0.72** | -0.14 | 0.04 | 0.04 |
| MEIme7 | -0.04 | 0.14 | **0.67** | -0.01 | 0.08 | -0.07 |
| MEIme8 | 0.12 | -0.23 | **0.66** | 0.02 | -0.02 | 0.09 |
| IDASdy61 | -0.03 | 0.16 | **-0.63** | 0.08 | -0.01 | 0.08 |
| MEIme14 | -0.02 | -0.07 | **0.63** | -0.07 | -0.03 | -0.03 |
| MEIme6 | -0.18 | -0.08 | **0.55** | -0.06 | 0.02 | -0.11 |
| MEIme3 | -0.16 | 0.01 | **0.49** | 0.00 | 0.06 | -0.12 |
| MEIme10 | -0.06 | -0.11 | **0.48** | -0.22 | -0.05 | -0.04 |
| IDASdy9 | 0.02 | 0.15 | **-0.47** | 0.13 | 0.07 | 0.22 |
| DASSd5 | 0.37 | 0.08 | **-0.39** | -0.03 | -0.12 | -0.04 |
| DASSd42 | 0.35 | -0.01 | **-0.38** | -0.03 | -0.15 | 0.01 |
| MEIsm11 | -0.21 | -0.12 | 0.29 | -0.14 | 0.29 | 0.07 |
| DASSs1 | -0.05 | 0.00 | 0.00 | **0.81** | -0.05 | 0.02 |
| DASSs27 | 0.04 | -0.12 | -0.12 | **0.76** | 0.03 | -0.03 |
| DASSs11 | 0.08 | 0.07 | 0.08 | **0.75** | -0.01 | 0.02 |
| DASSs6 | -0.07 | 0.17 | 0.00 | **0.67** | 0.04 | 0.09 |
| DASSs39 | 0.08 | 0.02 | -0.10 | **0.65** | -0.01 | -0.05 |
| DASSs18 | 0.13 | 0.03 | 0.00 | **0.65** | 0.08 | -0.06 |
| DASSs14 | 0.01 | 0.01 | -0.10 | **0.59** | -0.03 | -0.05 |
| DASSs29 | 0.12 | 0.26 | -0.10 | **0.40** | -0.10 | 0.05 |
| DASSs32 | 0.05 | 0.17 | -0.25 | **0.38** | 0.05 | 0.06 |
| DASSs35 | 0.11 | 0.26 | -0.19 | **0.37** | 0.03 | -0.10 |
| MEIsm26 | -0.07 | -0.08 | -0.04 | 0.04 | **0.80** | 0.09 |
| MEIsm25 | 0.12 | 0.08 | -0.01 | -0.03 | **0.78** | -0.08 |
| MEIsm27 | -0.03 | -0.04 | -0.04 | -0.03 | **0.76** | 0.14 |
| MEIsm20 | 0.03 | 0.17 | -0.06 | 0.05 | **0.62** | -0.05 |
| MEIsm23 | 0.05 | 0.21 | 0.10 | 0.02 | **0.59** | -0.14 |
| MEIsm19 | -0.13 | -0.08 | 0.02 | 0.16 | **0.56** | 0.03 |
| MEIsm24 | 0.02 | 0.09 | 0.00 | -0.15 | **0.55** | -0.04 |
| MEIsm21 | 0.10 | -0.07 | 0.01 | -0.04 | **0.54** | -0.03 |
| MEIme2 | -0.27 | 0.13 | 0.09 | 0.03 | **0.44** | -0.09 |
| MEIsm12 | -0.19 | 0.02 | 0.25 | -0.06 | **0.34** | 0.07 |
| IDASdy31 | 0.48 | 0.00 | -0.10 | 0.01 | -0.03 | **0.49** |
| IDASdy40 | 0.38 | 0.03 | -0.15 | 0.04 | -0.09 | **0.45** |
| IDASdy48 | 0.13 | 0.23 | -0.17 | 0.08 | -0.14 | **0.41** |
| IDASdy21 | 0.38 | 0.06 | -0.02 | 0.16 | -0.10 | **0.39** |

**S3.** Exploratory factor analysis item loadings on the 6 factors representing dimensions of anhedonia and related internalizing symptoms. Anh = anhedonia; Anx = anxiety; Cog = cognitive functioning; Dis = distress; Mot = motivation; Dys = dysphoria

## Supplementary Figures

**S0.** Percentage of recruited participants within each group by platform.


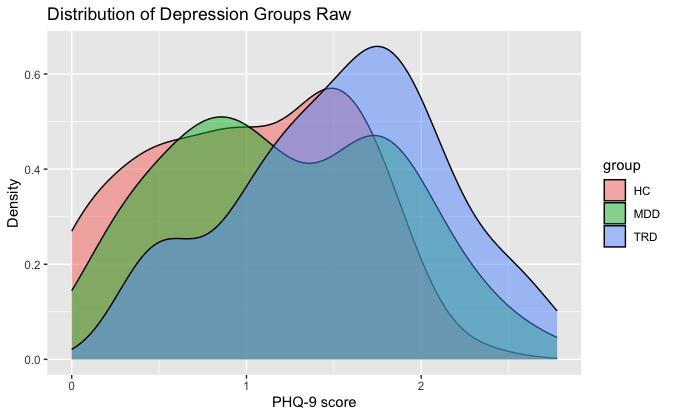


**S1.** Mean item score distribution on PHQ-9 of each group


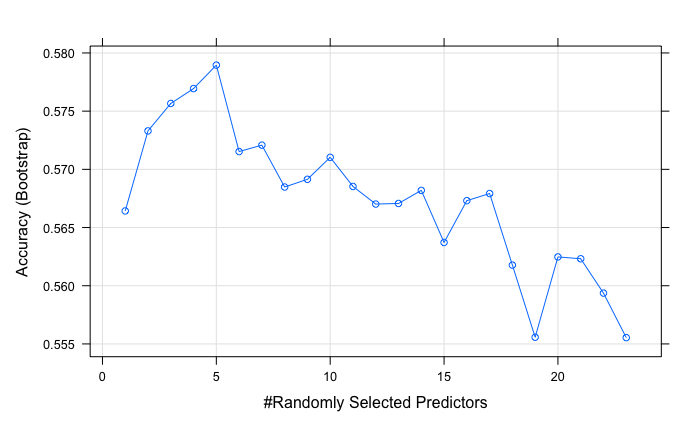


**S4.** Accuracy by number of predictors at each split for multiclass training data.
